# Combination of nivolumab with standard induction chemotherapy in children and adults with EBV-positive nasopharyngeal carcinoma: Protocol of a prospective multicenter phase 2 trial

**DOI:** 10.1007/s00106-023-01404-9

**Published:** 2024-01-12

**Authors:** Tristan Römer, Christian Vokuhl, Gundula Staatz, Felix M. Mottaghy, Hans Christiansen, Michael J. Eble, Beate Timmermann, Jens Peter Klussmann, Miriam Elbracht, Gabriele Calaminus, Martin Zimmermann, Tim H. Brümmendorf, Tobias Feuchtinger, Helena Kerp, Udo Kontny

**Affiliations:** 1 Division of Pediatric Hematology, Oncology and Stem Cell Transplantation, Medical Faculty, Center for Integrated Oncology Aachen Bonn Cologne Düsseldorf (CIO ABCD), RWTH Aachen University, Aachen, Germany; 2 Section of Pediatric Pathology, Department of Pathology, Center for Integrated Oncology Aachen Bonn Cologne Düsseldorf (CIO ABCD), University Hospital Bonn, Bonn, Germany; 3 Section of Pediatric Radiology, University Medical Center Mainz, Mainz, Germany; 4 Department of Nuclear Medicine, Medical Faculty, Center for Integrated Oncology Aachen Bonn Cologne Düsseldorf (CIO ABCD), RWTH Aachen University, Aachen, Germany; 5 Department of Radiotherapy and Radiation Oncology, Hannover Medical School, Hannover, Germany; 6 Department of Radiation Oncology, Center for Integrated Oncology Aachen Bonn Cologne Düsseldorf (CIO ABCD), RWTH Aachen University, Aachen, Germany; 7 Department of Particle Therapy, West German Proton Therapy Centre Essen (WPE), West German Cancer Centre (WTZ), University Hospital Essen, Essen, Germany; 8 Center for Integrated Oncology Aachen Bonn Cologne Düsseldorf (CIO ABCD), ENT Clinic of the University Hospital of Cologne, Cologne, Germany; 9 Institute for Human Genetics and Genomic Medicine, Medical Faculty, Center for Integrated Oncology Aachen Bonn Cologne Düsseldorf (CIO ABCD), RWTH Aachen University, Aachen, Germany; 10 Division of Pediatric Hematology and Oncology, Center for Integrated Oncology Aachen Bonn Cologne Düsseldorf (CIO ABCD), University Hospital Bonn, Bonn, Germany; 11 Division of Pediatric Hematology and Oncology, Hannover Medical School, Hannover, Germany; 12 Department of Hematology, Oncology, Hemostaseology, Stem Cell Transplantation, Medical Faculty, Center for Integrated Oncology Aachen Bonn Cologne Düsseldorf (CIO ABCD), RWTH Aachen University, Aachen, Germany; 13 Division of Pediatric Hematology and Oncology, Department of Pediatrics and Adolescent Medicine, Medical Center, Faculty of Medicine, University of Freiburg, Freiburg, Germany; 14 Pediatric Research Network gGmbH, University of Duisburg-Essen, Essen, Germany; 15 Sektion Pädiatrische Hämatologie, Onkologie und Stammzelltransplantation, Klinik für Kinder- und Jugendmedizin, Uniklinik RWTH Aachen, Pauwelsstraße 30, 52074 Aachen, Germany

**Keywords:** Adolescents, Interferon, Immune checkpoint-inhibitor, Radiotherapy, PD-1, Jugendliche, Interferon, Immuncheckpointinhibitor, Strahlentherapie, PD-1

## Abstract

**Background:**

Treatment of Epstein-Barr virus(EBV)-positive nasopharyngeal carcinoma (NPC) with cisplatin/5-fluorouracil (5-FU) induction chemotherapy, followed by radiochemotherapy and subsequent interferon‑β, has yielded high survival rates in children, adolescents, and young adults. A previous study has shown that reduction of radiation dose from 59.4 to 54.0 Gy appears to be safe in patients with complete response (CR) to induction chemotherapy. As immune checkpoint-inhibitors have shown activity in NPC, we hypothesize that the addition of nivolumab to standard induction chemotherapy would increase the rate of complete tumor responses, thus allowing for a reduced radiation dose in a greater proportion of patients.

**Methods:**

This is a prospective multicenter phase 2 clinical trial including pediatric and adult patients with their first diagnosis of EBV-positive NPC, scheduled to receive nivolumab in addition to standard induction chemotherapy. In cases of non-response to induction therapy (stable or progressive disease), and in patients with initial distant metastasis, treatment with nivolumab will be continued during radiochemotherapy. Primary endpoint is tumor response on magnetic resonance imaging (MRI) and positron emission tomography (PET) after three cycles of induction chemotherapy. Secondary endpoints are event-free (EFS) and overall survival (OS), safety, and correlation of tumor response with programmed cell death ligand 1 (PD-L1) expression.

**Discussion:**

As cure rates in localized EBV-positive NPC today are high with standard multimodal treatment, the focus increasingly shifts toward prevention of late effects, the burden of which is exceptionally high, mainly due to intense radiotherapy. Furthermore, survival in patients with metastatic disease and resistant to conventional chemotherapy remains poor. Primary objective of this study is to investigate whether the addition of nivolumab to standard induction chemotherapy in children and adults with EBV-positive NPC is able to increase the rate of complete responses, thus enabling a reduction in radiation dose in more patients, but also offer patients with high risk of treatment failure the chance to benefit from the addition of nivolumab.

**Trial registration:**

EudraCT (European Union Drug Regulating Authorities Clinical Trials Database) No. 2021-006477-32.

## Abstract (de)

**Hintergrund:**

Bei der Behandlung des Epstein-Barr Virus(EBV)-positiven Nasopharynxkarzinoms (NPC) wurden durch Induktionschemotherapie mit Cisplatin/5-Fluorouracil (5-FU), gefolgt von Radiochemotherapie und anschließend Interferon‑β als Erhaltungstherapie, hohe Überlebensraten im Kindes‑, Jugend- und jungen Erwachsenenalter erzielt. Bei Patient*innen mit komplettem Ansprechen (CR) auf die Induktionstherapie konnte dabei die Strahlendosis von 59,4 auf 54,0 Gy reduziert werden. Aufgrund nachgewiesener Aktivität von Immuncheckpointinhibitoren beim NPC verfolgen die Autor*innen den Ansatz, durch Hinzunahme von Nivolumab zur Standard-Induktionschemotherapie die CR-Rate weiter zu verbessern, um mehr Patient*innen die reduzierte Strahlendosis anbieten zu können.

**Methodik:**

In dieser prospektiven multizentrischen Phase-II-Studie, in die sowohl Kinder als auch Erwachsene mit Erstdiagnose eines EBV-positiven NPC eingeschlossen werden können, wird Nivolumab zusätzlich zur Standard-Induktionstherapie verabreicht. Bei Nichtansprechen (stabile oder progrediente Erkrankung), und bei Vorhandensein initialer Fernmetastasen, wird die Therapie mit Nivolumab während der Radiochemotherapie fortgeführt. Primärer Endpunkt ist das Ansprechen in der Magnetresonanztomographie (MRT) und Positronenemissionstomographie (PET) nach 3 Zyklen Induktionstherapie. Sekundäre Endpunkte sind ereignisfreies (EFS) und Gesamtüberleben (OS), Verträglichkeit und Korrelation des Tumoransprechens mit der „programmed cell death ligand 1“(PD-L1)-Expression.

**Schlussfolgerung:**

Da heutzutage durch multimodale Standardbehandlung hohe Heilungsraten beim lokalisierten EBV-positiven NPC erreicht werden, richtet sich der Fokus zunehmend auf die Prävention therapiebedingter Spätfolgen, deren Ausmaß vor allem infolge der intensiven Strahlentherapie beträchtlich ist. Darüber hinaus weisen Patient*innen mit metastasierter oder gegenüber konventioneller Chemotherapie refraktärer Erkrankung weiterhin eine schlechte Prognose auf. Primäres Ziel der vorliegenden Studie ist zu untersuchen, ob die Hinzunahme von Nivolumab zur Standard-Induktionschemotherapie bei Kindern und Erwachsenen mit EBV-positivem NPC die CR-Rate zu verbessern vermag, wodurch eine Reduktion der Strahlendosis bei mehr Patient*innen möglich wäre. Zusätzlich bietet die Studie Patient*innen mit hohem Risiko für ein Therapieversagen die Möglichkeit, von der zusätzlichen Behandlung mit Nivolumab zu profitieren.

**Registrierung:**

EudraCT (European Union Drug Regulating Authorities Clinical Trials Database) Nr. 2021-006477-32.

## Administrative information

Note: the numbers in curly brackets in this protocol refer to SPIRIT (Standard Protocol Items: Recommendations for Interventional Trials) checklist item numbers. The order of the items has been modified to group similar items (see http://www.equator-network.org/reporting-guidelines/spirit-2013-statement-defining-standard-protocol-items-for-clinical-trials/). For administrative information, see Table 1.

**Table 1:** Administrative information

Title {1}	Nivolumab in combination with cisplatin and 5‑fluorouracil as induction therapy in children and adults with EBV-positive nasopharyngeal carcinoma
Trial registration {2a and 2b}	EudraCT (European Union Drug Regulating Authorities Clinical Trials Database) No. 2021-006477-32; DRKS (German Clinical Trials Register): DRKS00027098; ClinicalTrials.gov: NCT06019130
Protocol version {3}	Version 1.3 Final, March 3, 2023
Funding {4}	This investigator-initiated clinical trial is funded by the Deutsche Krebshilfe (German Cancer Aid) as is the Center for Integrated Oncology at Aachen-Bonn-Cologne-Düsseldorf (CIO^ABCD^). Bristol-Myers Squibb provides support for this trial by providing nivolumab
Author details {5a}	*Coordinating (chief) investigator:* Udo Kontny
	*Assistant investigator:* Tristan Römer
	Both: Division of Pediatric Hematology, Oncology and Stem Cell Transplantation, Medical Faculty, RWTH Aachen University, Aachen, Germany, Center for Integrated Oncology Aachen Bonn Cologne Düsseldorf (CIO ABCD)
	**Trial reference centers**
	*Pathology*
	Christian Vokuhl, Section of Pediatric Pathology, Department of Pathology, University Hospital Bonn, Bonn, Germany, Center for Integrated Oncology Aachen Bonn Cologne Düsseldorf (CIO ABCD)
	*Radiology*
	Gundula Staatz, Section of Pediatric Radiology, University Medical Center Mainz, Mainz, Germany
	*Nuclear Medicine*
	Felix M. Mottaghy, Department of Nuclear Medicine, Medical Faculty, RWTH Aachen University, Aachen, Germany, Center for Integrated Oncology Aachen Bonn Cologne Düsseldorf (CIO ABCD)
	*Radiotherapy*
	Hans Christiansen, Department of Radiotherapy and Radiation Oncology, Hannover Medical School, Hannover, Germany
	Michael J. Eble, Department of Radiation Oncology, RWTH Aachen University, Aachen, Germany, Center for Integrated Oncology Aachen Bonn Cologne Düsseldorf (CIO ABCD)
	Beate Timmermann, Department of Particle Therapy, University Hospital Essen, West German Proton Therapy Centre Essen (WPE), West German Cancer Centre (WTZ), Essen, Germany
	*Head and Neck Surgery*
	Jens Peter Klussmann, ENT Clinic of the University Hospital of Cologne, Cologne, Germany, Center for Integrated Oncology Aachen Bonn Cologne Düsseldorf (CIO ABCD)
	*Pharmacogenomics*
	Miriam Elbracht, Institute for Human Genetics and Genomic Medicine, Medical Faculty, RWTH Aachen University, Aachen, Germany, Center for Integrated Oncology Aachen Bonn Cologne Düsseldorf (CIO ABCD)
	*Patient-reported Outcome*
	Gabriele Calaminus, Division of Pediatric Hematology and Oncology, University Hospital Bonn, Bonn, Germany, Center for Integrated Oncology Aachen Bonn Cologne Düsseldorf (CIO ABCD)
	*Biostatistics*
	Martin Zimmermann, Division of Pediatric Hematology and Oncology, Hannover Medical School, Hannover, Germany
	**Additional steering committee members**
	Tim H. Brümmendorf, Department of Hematology, Oncology, Hemostaseology, Stem Cell Transplantation, Medical Faculty, RWTH Aachen University, Aachen, Germany, Center for Integrated Oncology Aachen Bonn Cologne Düsseldorf (CIO ABCD)
	Tobias Feuchtinger, Division of Pediatric Hematology and Oncology, Department of Pediatrics and Adolescent Medicine, Medical Center, Faculty of Medicine, University of Freiburg, Freiburg, Germany
	**Project management and monitoring:**
	Helena Kerp, Pediatric Research Network gGmbH, University of Duisburg-Essen, Essen, Germany
Name and contact information for the trial sponsor {5b}	German Society for Pediatric Oncology and Hematology (GPOH) gGmbH, registered at: Chausseestraße 128/129, 10115 Berlin, Germany; Sponsor office: Holsterhauser Platz 2, 45147 Essen, Germany
Role of sponsor {5c}	n/a: The funders have no role in study design; collection, management, analysis, and interpretation of data; writing of the report; and the decision to submit the report for publication, and they do not have ultimate authority over any of these activities

## Introduction

### Background and rationale {6a}

NPC is a highly malignant tumor arising from epithelial cells of the nasopharynx. Its incidence shows significant geographic variation, with the highest rates in Southeast Asia [1]. In Western Europe, NPC is a rare cancer with an age-standardized incidence rate of 0.5/100,000 per year [2]. In children, adolescents, and young adults worldwide, and in adults from endemic regions, NPC is predominantly of undifferentiated histology, consistently associated with detection of EBV in tumor cells and a high degree of lymphomononuclear cell infiltration. About 90% of patients have locoregionally advanced disease without overt distant metastases at the time of diagnosis, showing overall survival rates of about 90% with state-of-the-art treatment. On the other hand, patients with metastatic or relapsed disease have much poorer chances for cure with long-term survival rates below 20% [3].

As NPC is radiosensitive and usually not amenable to complete surgical excision, radiotherapy has traditionally been the treatment of choice. Radiation doses of around 70 Gy to the primary tumor and involved lymph nodes are considered standard in adults [4]. In children and adolescents, NPC has also shown high chemosensitivity, with response rates to cisplatin/5-FU induction of more than 90% [5, 6]. Therefore, radiation doses in children have traditionally been lower than in adults, with excellent EFS rates of between 79% and 92% documented by study groups in Germany, Italy, and North America [5, 6; 7]. Notably, the German Society of Pediatric Oncology and Hematology (GPOH) study, which used the lowest radiation dose (54.0–59.4 Gy), achieved the highest EFS and OS of 92% and 97%, respectively [5]. These data were recently confirmed in an extended follow-up analysis, also showing that the few relapses almost exclusively occurred at distant sites and within 2 years of end of treatment [8]. A unique hallmark of the GPOH studies is the application of interferon-beta (IFN-β) for 6 months after completion of radiochemotherapy, possibly contributing to the high survival and low relapse rates by improving systemic disease control. As NPC is curable today for the majority of patients, the main focus shifts toward prevention of long-term toxicities, especially in the most vulnerable group of young patients. Frequency and severity of long-term toxicities are known to be mainly determined by the intensity of radiotherapy, and thus a reduction of the radiation dose in patients with good response to induction chemotherapy could be a means of reducing the burden of late effects. In the prospective multicenter study *NPC-2003* of the GPOH, patients with complete response to induction chemotherapy received a reduced dose of 54.0 Gy to the primary tumor bed compared to 59.4 Gy for patients with residual tumor, and none of these patients relapsed during 7 years of follow-up [5, 8].

An attractive and less toxic option to increase efficacy of medical treatment for NPC could be the addition of an anti-programmed cell death 1 (PD-1) antibody to induction chemotherapy. The NPC cells express the immune effector cell inhibiting programmed cell death ligand 1 (PD-L1) in about 85% of tumors [9], and tumor-infiltrating cytotoxic T‑lymphocytes (CTLs) show high expression of PD‑1 [10]. It has been demonstrated that high PD-1/PD-L1 expression significantly correlates with worse disease-free survival in NPC [10–12]. Therefore, blocking the PD-1/PD-L1 interaction is believed to substantially increase cellular antitumor activity against NPC cells, as already observed in other malignancies, for example, non-small-cell lung cancer (NSCLC; [13, 14]). In two phase 2 trials in which patients with refractory NPC were treated with the anti-PD1 antibodies pembrolizumab or nivolumab as monotherapy, overall response rates of 26% and 20.5%, respectively, were observed [15, 16]. In the *Keynote-048* trial, 882 patients with locally incurable refractory or metastatic head and neck squamous cell carcinoma (HNSCC) were randomized to receive either pembrolizumab or cetuximab, plus six cycles of cisplatin/5-FU, showing a significantly improved OS in the pembrolizumab arm for patients with a PD-L1 Combined Positive Score (CPS) ≥ 1 [17]. More recently, 263 patients with metastatic or recurrent NPC were randomly assigned to receive the anti-PD1 antibody camrelizumab or placebo, plus gemcitabine/cisplatin, and progression-free survival (PFS) was significantly longer in the camrelizumab vs. placebo arm [18]. In children, pembrolizumab has already been licensed for treatment-refractory Hodgkin lymphoma in children ≥ 3 years of age. Data on the safety and efficacy of the anti-PD‑1 antibodies nivolumab and pembrolizumab in children are available from two large multicenter phase 1/2 trials, the *ADVL1412* and the *Keynote-051* trial. These trials demonstrated encouraging antitumor activity and a favorable toxicity profile also in the pediatric population [19, 20].

### Objectives {7}

The objectives are to evaluate the efficacy and safety of adding the immune checkpoint-inhibitor nivolumab to standard induction chemotherapy for pediatric and adult patients with EBV-positive NPC. If the addition of nivolumab would increase the rate of complete tumor responses to induction therapy, subsequent radiotherapy could be dose-de-escalated in more patients, potentially reducing the burden of severe late effects in this population. Furthermore, patients with a high risk of treatment failure, that is, patients with metastatic disease and those not responsive to conventional chemotherapy, could substantially benefit from the addition of nivolumab to standard treatment.

### Trial design {8}

This is a prospective multicenter single-arm phase 2 clinical trial.

## Methods: participants, interventions, and outcomes

### Study setting {9}

This study is planned to be carried out at 36 study centers in Germany including 33 university hospitals and three large tertiary non-academic centers. An updated list of open centers is available at npc@ukaachen.de or from the coordinating investigator.

### Eligibility criteria {10}

#### Inclusion criteria

- Histologically confirmed new diagnosis of NPC according to the current World Health Organization (WHO) classification in children and adolescents aged between 3 and 17 years, *or* histologically confirmed new diagnosis of EBV-positive NPC, WHO type II or III, in individuals aged ≥ 18 years
- Stage II or higher in patients ≤ 25 years, stage III and IV in patients > 25 years (according to American Joint Committee on Cancer [AJCC], 8th edition)
- Measurable disease by MRI per Response Evaluation Criteria in Solid Tumors (RECIST) 1.1 criteria
- Sufficient tumor tissue to be sent for central review, including PD-L1 staining, either as one or two full blocks (preferred) or a minimum of 25 slides, obtained from core biopsy, punch biopsy, excisional biopsy, or surgical specimen
- Written informed consent of patient and legal guardian (if patient not ≥ 18 years)

#### Exclusion criteria

- Newly diagnosed NPC stage I in all patients, stage II in patients > 25 years
- Recurrent NPC
- NPC diagnosed as second malignancy after preceding chemotherapy and/or radiotherapy
- Prior chemotherapy and/or radiotherapy
- Other active malignancy
- Prior treatment with an anti-PD‑1, anti-PD-L1, anti-PD-L2, anti-cytotoxic T‑lymphocyte-associated antigen (CTLA)‑4 antibody, or any other antibody or drug specifically targeting T‑cell co-stimulation or checkpoint pathways
- Investigational drug within 30 days prior to inclusion
- Enrolment in another clinical trial
- Prior organ allograft or allogenic bone marrow transplantation
- Active, known or suspected autoimmune disease; type I diabetes mellitus, hypothyroidism only requiring hormone replacement, skin disorders (such as vitiligo, psoriasis, or alopecia) not requiring systemic treatment, or conditions not expected to recur in the absence of an external trigger are permitted for enrollment
- Condition requiring systemic treatment with either corticosteroids (> 10 mg daily prednisone equivalent) or other immunosuppressive medications within 14 days before start of therapy; inhaled or topical steroids, and adrenal replacement steroid doses > 10 mg daily prednisone equivalent, are permitted in the absence of active autoimmune disease
- Any positive test for hepatitis B virus or hepatitis C virus indicating acute or chronic infection
- Known history of testing positive for human immunodeficiency virus (HIV) or known acquired immunodeficiency syndrome (AIDS)
- Inadequate hematological, renal, or hepatic function defined by any of the following screening laboratory values:
  - White blood cells (WBC) < 2000/μL
  - Neutrophils < 1500/μL
  - Platelets < 100 × 10^3^/μL
  - Hemoglobin < 9.0g/dL
  - Creatinine > 1.5 x upper limit of normal (ULN) or creatinine clearance < 50 mL/min (using the Cockcroft–Gault formula or Schwartz formula in patients < 18 years)
  - Aspartate aminotransferase (AST)/alanine aminotransferase (ALT) > 3 x ULN (> 5 x ULN if liver metastases)
  - Total bilirubin > 1.5 x ULN (except patients with Gilbert syndrome who must have a total bilirubin level ≥ 3.0 x ULN)
- Hearing loss > 20 dB loss at 3 kHz, due to an inner ear disorder and not caused by tumor burden
- History of allergy or hypersensitivity to platinum-containing compounds or other study drug components
- Clinically significant, uncontrolled heart disease (including history of any cardiac arrhythmias, e.g., ventricular, supraventricular, nodal arrhythmias, or conduction abnormality within 12 months of screening)
- Vaccinated with live attenuated vaccines within 4 weeks of the first dose of the study drug
- Inadequate performance status (Karnofsky score < 60 for patients ≥ 16 years, Lansky score < 60 for patients < 16 years)

#### Who will take informed consent? {26a}

Participation in the trial is voluntary for all patients. All patients and/or parent/legal guardians are informed by the investigator verbally and in writing about the aim, treatment, anticipated benefits, and potential risks of taking part in the trial, and that they are completely free to refuse to take part or withdraw from the trial at any time without giving a reason. A patient information sheet will be handed out and there will be sufficient time for the patient and/or the parent/legal guardian to decide on whether to take part in the trial as well as for clarification of any questions. As the trial includes children and adolescents, written consent/assent will be obtained from the patient whenever possible (as appropriate according to age and national legislation), and there is a section in the parent informed consent form where assent can be obtained from the patient. For those children who are not able to read, write, or understand the content, the investigator will explain the trial and obtain verbal assent that will be documented in the patient’s medical record.

#### Additional consent provisions for collection and use of participant data and biological specimens {26b}

All patients and/or parent/legal guardians are informed by the investigator about the transmission and use of data and biological specimens (tumor tissue, blood, saliva) collected. Studies on biological specimens can be denied separately by patients and/or parent/legal guardians in the consent form.

## Interventions

### Explanation for the choice of comparators {6b}

n/a: This is a non-randomized interventional trial without comparator arms. Efficacy of the investigational drug will be evaluated in relation to historical controls, since the rarity of the patient population does not allow for fully powered randomized comparison. The rationale for use of the investigational drug is depicted in the section “Background and rationale.”

### Intervention description {11a}

Patients are scheduled to receive nivolumab in addition to standard induction chemotherapy, which is gemcitabine/cisplatin for patients with distant metastases and age > 25 years, and cisplatin/5-FU for all others. In the case of non-response to induction therapy (i.e., stable or progressive disease), and in patients with initial distant metastases, treatment with nivolumab will be continued throughout subsequent radiochemotherapy. Nivolumab is given in a single dose of 4.5 mg/kg (max. 360 mg) every 3 weeks for a maximum of three applications in patients with non-metastatic disease responding to induction chemotherapy, or six applications in all others (occasionally seven applications in patients with metastatic disease responding to induction therapy who are eligible for a fourth cycle of induction chemotherapy).

**Fig. 1:**
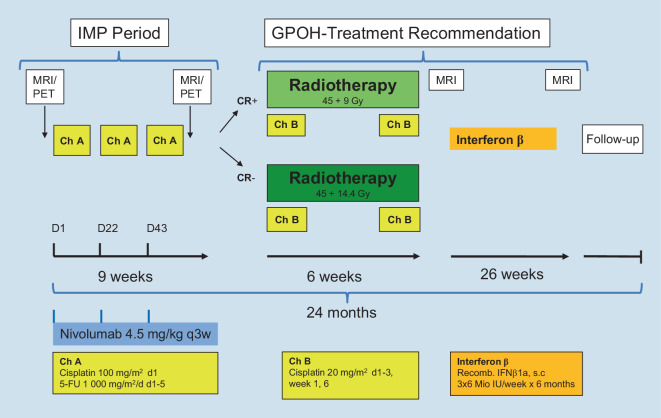
Intervention flowchart for patients ≤ 25 years with non-metastatic nasopharyngeal carcinoma and complete response or partial response after induction therapy. *IMP* investigational medical product, *GPOH* German Society of Pediatric Oncology and Hematology, *MRI* magnetic resonance imaging, *PET* positron emission tomography, *IFN-β* Interferon β, *5-FU* 5-fluorouracil, *Ch A* chemotherapy cycle A, *Ch B* chemotherapy cycle B, *D* day, *q3w* every three weeks, *CR* complete remission

According to age, response to induction therapy, and presence or absence of initial distant metastases, patients are allocated to one of five treatment strata, which are described in detail in the following section:

Children, adolescents, and young adults ≤ 25 years:

1. Non-metastatic NPC with CR or PR after induction therapy
  Induction therapy will comprise three cycles of cisplatin/5-FU (chemotherapy cycle A [*Ch A*]) in combination with nivolumab at 3‑week intervals. Nivolumab is given in a single dose of 4.5 mg/kg (max. 360 mg), followed by cisplatin 100 mg/m^2^ as continuous infusion over 6 h, and then 5‑FU 1000 mg/m^2^/day as continuous infusion over 5 days.
  Treatment with nivolumab is ended after a total of three doses together with induction chemotherapy, when CR or partial response (PR) is noted in MRI and PET-CT/-MRI on day 17–22 of cycle 3. Concomitant chemoradiotherapy (CCRT) should begin approximately 4 weeks after the start of the last induction chemotherapy cycle. Patients with PR will receive a radiation dose of 45.0 Gy to the primary target volume (PTV1; including elective irradiated lymph node levels) with a boost to 59.4 Gy to PTV2 (depending on residual disease), using standard fractionation of 5 × 1.8 Gy per week. In patients with CR, the radiation dose including boost will be reduced to 54.0 Gy (45.0 + boost 9.0 Gy). Use of an image-guided high-precision radiation therapy with image fusion and 3D-computerized tomography (CT) based planning is mandatory. High-energy photon linear accelerators or proton technologies can be used for beam production. For radiosensitization, cisplatin 20 mg/m^2^/day is given on days 1–3 of the first and last week of radiotherapy (two cycles of chemotherapy cycle B [*Ch B*], total cisplatin dose 120 mg/m^2^). In patients with ototoxicity grade > 2 or creatinine clearance < 70 ml/min/1.73m^2^, cisplatin should be replaced by carboplatin.
  Maintenance therapy with interferon (IFN)-β 1a is started the week after the end of CCRT, if starting criteria are met (good general health, no severe depression and/or suicidal ideation, granulocytes > 750/µL, thrombocytes > 50,000/µL). IFN-β is injected subcutaneously at a dose of 22 µg (= 6 million international units [IU]) three times a week. Interferon maintenance will be applied over 6 months (26 weeks).
  The study flowchart for patients ≤ 25 years with non-metastatic NPC and CR or PR after induction therapy is depicted in Fig. 1.
2. Non-metastatic NPC with stable (SD) or progressive disease (PD) after induction therapy *or* patients with distant metastases at initial diagnosis
  Patients will receive induction chemotherapy with cisplatin/5-FU (*Ch A*) and nivolumab, but application of nivolumab every 3 weeks is continued throughout subsequent radiochemotherapy for a total of six doses. Patients with metastatic disease responding to induction therapy (PR or CR) are eligible for a fourth cycle of chemotherapy, and thus these patients will receive a total of seven nivolumab doses.
  CCRT is applied as described above, with all patients receiving a local radiation dose including a boost of 59.4 Gy. Oligometastases (e.g., bones, liver, lung) can also be irradiated with an individual dose-/volume-concept in accordance to the metastatic site and the standards/expertise of the respective radio-oncological center.
  Interferon maintenance is the same as for patients with non-metastatic disease responding to induction therapy (see above).

**Fig. 2:**
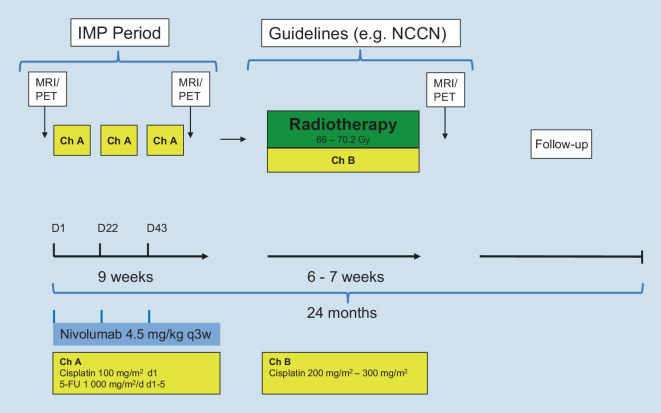
Intervention flow chart for patients > 25 years with non-metastatic nasopharyngeal carcinoma and complete response or partial response after induction therapy. *IMP* investigational medical product, *NCCN* National Comprehensive Cancer Network, *MRI* magnetic resonance imaging, *PET* positron emission tomography, *5-FU* 5-fluorouracil, *Ch A* chemotherapy cycle A, *Ch B* chemotherapy cycle B, *D* day, *q3w* every three weeks

**Fig. 3:**
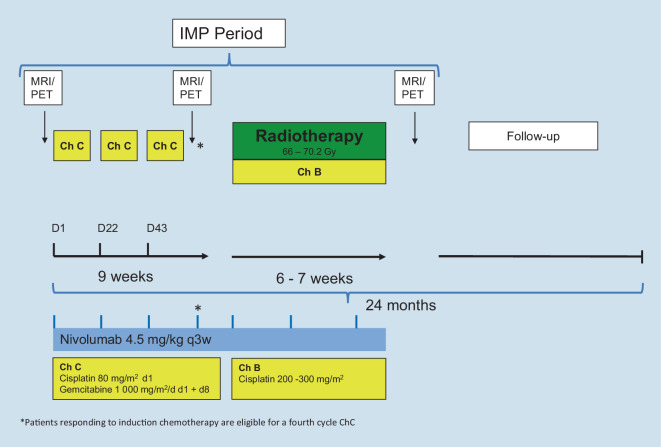
Intervention flowchart for patients > 25 years with initial distant metastases. *IMP* investigational medical product, *MRI* magnetic resonance imaging, *PET* positron emission tomography, *Ch B* chemotherapy cycle B, *Ch C* chemotherapy cycle C, *D* day, *q3w* every three weeks

Adults > 25 years:

3. Non-metastatic NPC with CR or PR after induction therapy
  Induction chemotherapy and nivolumab treatment will not differ from the regimen in patients ≤ 25 years, with a total of three doses of nivolumab given synchronously to cisplatin/5-FU induction chemotherapy (three cycles *Ch A*). However, the radiation dose should be given according to published guidelines for adults, with a recommended dose range of 66–70.2 Gy [4]. Also, higher cisplatin doses totaling between 200 and 300 mg/m^2^ are recommended for CCRT in adults and should be applied according to local practice (*Ch B*). Interferon maintenance is not part of the study treatment plan for adults > 25 years due to lack of sufficient clinical data.
  The study flowchart for adult patients > 25 years with non-metastatic NPC and CR or PR after induction therapy is shown in Fig. 2.
4. Non-metastatic NPC with SD or PD after induction therapy
  As for patients ≤ 25 years with non-response to induction chemotherapy, application of nivolumab will be extended throughout radiochemotherapy for a total of six doses. CCRT is applied as described for adults > 25 years with CR or PR (see above).
5. Distant metastases at initial diagnosis (local therapy in curative intention)
  Adults > 25 years with distant metastases at diagnosis will receive three cycles of gemcitabine/cisplatin (*Ch C*). Nivolumab is given during induction chemotherapy and throughout radiochemotherapy, as described for patients with non-response (see above). Patients responding to induction therapy (PR or CR) are eligible for a fourth cycle of chemotherapy, so these patients will receive a total of seven nivolumab doses. CCRT, including irradiation of distant metastases, is applied according to published guidelines for adults (see above).
  Flowchart for adult patients > 25 years with initial distant metastases is shown in Fig. 3.

#### Criteria for discontinuing or modifying allocated interventions {11b}

The following rules for treatment discontinuation in the individual patient will apply:

- Patient’s or parent/legal guardian’s request to stop treatment
- Withdrawal of consent to data collection
- Confirmed radiographic disease progression during induction therapy
- Any clinical adverse event (AE), laboratory abnormality, or intercurrent illness that, in the opinion of the investigator, indicates that continued participation in the study is not in the best interest of the patient
- Less than one dose of nivolumab during induction therapy
- Loss of ability to freely provide consent through imprisonment or involuntarily incarceration for treatment of either a psychiatric or physical illness
- Non-compliance
- Pregnancy
- For additional protocol-specified reasons for treatment discontinuation, please refer to the full protocol that is provided by the coordinating investigator

Premature study termination is indicated by the following events:

- Unjustifiable risk and/or toxicity in risk–benefit analysis (decision taken by sponsor or its representative), e.g., when AEs occur, unknown to date in terms of their nature, severity, duration, or frequency in relation to the current established safety profile (substantial changes in risk–benefit considerations), and therefore medical and/or ethical reasons affect the continued performance of the trial or for a specific patient cohort or specific the subgroup
- New scientific evidence becomes available during the study that could affect the patient’s safety (benefit–risk analysis no longer positive), e.g., new insights from other clinical trials
- Request of the sponsor or regulatory agency, e.g., as a consequence of monitoring or inspection
- In the case of difficulties in the recruitment of the planned number of participants in the indicated time (insufficient recruitment rate)
- Permanently unavailability of an investigational medicinal product (IMP) or withdrawal of the license to manufacture or of the permission to import an IMP
- The maximal sum insured cannot be adjusted, although required

The decision for premature study termination will be made by the sponsor or its representatives in consultation with the data monitoring and steering committee. If there is an imminent risk of harm to the enrolled patients, the sponsor or its representatives will immediately discontinue the enrolment and verify the applicability of the aforementioned criteria.

#### Strategies to improve adherence to interventions {11c}

Comprehensive briefing at pre-study and initiation visits and continuous monitoring are measures to improve adherence. Sites are provided with an investigator site file, including all essential documents and forms for data collection.

Compliance for IMP treatment will be monitored by representatives of the sponsor as specified in the risk management plan and the monitoring plan. The prescription and use of the IMP is recorded on the treatment case report form (CRF).

### Relevant concomitant care permitted or prohibited during the trial {11d}

Participants are permitted to use topical, ocular, intra-articular, intranasal, and inhalational corticosteroids (with minimal systemic absorption). Adrenal replacement steroid doses > 10 mg daily prednisone equivalent in the absence of active autoimmune disease, and a brief (less than 3 weeks) course of corticosteroids for prophylaxis (e.g., contrast dye allergy) or for treatment of non-autoimmune conditions (e.g., delayed-type hypersensitivity reaction caused by a contact allergen) are also permitted.

The following medications or measures are prohibited during the study (unless utilized to treat a drug-related AE):

- Immunosuppressive agents within 14 days of starting study treatment
- Immunosuppressive doses of systemic corticosteroids (> 10 mg daily prednisone equivalent), except as stated above
- Any concurrent anti-neoplastic therapy (i.e., chemotherapy, hormonal therapy, immunotherapy, radiotherapy other than specified in this protocol)
- Surgical resection of tumor
- Any botanical preparation (e.g., herbal supplements or traditional Chinese medicines) intended to treat the disease under study or to provide supportive care

Caution should be used regarding the use of herbal medications as there may be yet unknown interactions with nivolumab. Discontinuation of the use of herbal medications prior to study enrolment is encouraged.

### Provisions for post-trial care {30}

For all patients participating in this trial, an insurance covering trial-related harms is contracted.

No specific post-trial care will be performed. All patients will return to their standard medical care after the trial.

## Outcomes {12}

### Primary outcome

Primary endpoint is CR rate after induction therapy with cisplatin/5-FU in combination with nivolumab, defined as the proportion of participants achieving CR on MRI *and* PET-CT/-MRI between day 17 and 22 after starting the third cycle of induction therapy. Tumor response will be assessed by central review of MRI and PET-CT/-MRI using RECIST 1.1 criteria. The finding of CR after induction therapy enables reduction of the radiation dose from 59.4 to 54.0 Gy in patients ≤ 25 years with advanced locoregional disease, according to the study protocol. In patients with SD or PD, imaging assessment will be repeated 12 weeks after the end of CCRT.

### Secondary outcomes

The following secondary endpoints are obtained:

- Event-free and overall survival
- Safety of nivolumab in combination with standard induction chemotherapy in children and adults with NPC
- Safety of nivolumab in combination with CCRT in children and adults with NPC not responding to induction therapy or with initial distant metastases
- Correlation of efficacy of nivolumab with PD-L1 expression in tumor tissue (as measured by CPS [21])

Safety of the study treatment is assessed by monitoring and recording of all AEs as per common terminology criteria for adverse events (CTCAE) v5.0 and serious adverse events (SAEs). For this purpose, regular study visits with measurement of vital signs, performance of physical examinations, laboratory evaluation for hematology, blood chemistry, and urine values as well as audiometry will be performed.

### Participant timeline {13}

The entire trial will last 60 months. The period for accrual of patients will last 36 months, with the first patient enrolled in January 2023. The trial report is planned to be completed by 2028.

For the time schedule of patient enrolment, visits, and assessments, see Table 2.

**Table 2:** Visit schedule with assessments at each visit

Phase	Pre-study	Treatment					Off treatment	Follow-up
*Study period*	*Screening*	*Chemo* *Cycle 1*	*Chemo* *Cycle 2*	*Chemo* *Cycle 3*		*Radiotherapy*^*1*^		
*Visit no.*	*1*	*2*	*3*	*4*	*5*	*5add*^*1*^	*6*	
*Activity*		*Baseline*			*Response evaluation*	*Response evaluation*^*1*^		*Disease status*
*Visit window* *±* *calendar days*^*19*^	*Day -14–0*	*Day 1*	*Day 0–1*	*Day 0–1*	*Day 17–22*	*12 weeks after last dose of radiotherapy*^*1*^	*100 days* *±3 days after last dose of nivolumab*	*Every 3 months until 2 years from enrolment*
Written informed consent	X							
Inclusion/exclusion criteria	X	X						
Tumor tissue sample^2^	X							
Demographics and medical history	X							
Concomitant medication	X	X	X	X	X	X	X	
Physical examination	X	X	X	X	X	X	X	
Vital signs^3^	X	X	X	X	X	X	X	
Height (cm)	X						X	
Weight (kg)	X	X	X	X	X	X	X	
Performance status^4^	X	X	X	X	X	X	X	
12-lead ECG^5^	X						X	
Echocardiography^5^	X						X	
Creatinine-clearance (24‑h urine)^5^	X		X	X			X	
Audiometry^5,18^	X		X	X	X		X	
Dental exam^5^	X						X	
Ophthalmological exam^5^	X						X	
Pregnancy test^5,6^ (WOCBP only)	X	X	X	X			X	
Laboratory tests^5,7^	X	X	X	X	X	X	X	
Hepatitis screening^5,8^	X						X	
HIV testing^5^	X						X	
DPD genetics^5,9^	X							
EBV-PCR^5,10^		X^11^	X		X	X	X	X
Biomarkers (blood)^12^		X^11^	X	X	X	X	X	
Biomarkers (saliva)^12^		X^11^			X	X	X	
MRI head/neck^5,13^	X				X	X		X
PET-CT or PET-MRI^5,14^	X				X	X		
MRI abdomen^5,15^	X							
Study drug administration		X	X	X				
AE evaluation^16^			X	X	X	X	X	
Disease and event status								X
PROs Assessment	X				X		X	X^17^

^1^Only for patients with metastatic disease at diagnosis or non-metastatic disease with SD/PD at visit 5; this visit can be combined with visit 6, if calculated time points for visits 5add and 6 are not > 1 week apart

^2^Tumor tissue to be sent for central review, including PD-L1 staining is mandatory. Sufficient tumor tissue should be submitted either 1–2 full blocks (preferred) or a minimum of 25 slides, obtained from core biopsy, punch biopsy, excisional biopsy or surgical specimen. Contact the Study Director if sufficient tissue is not available

^3^*Vital signs* will include temperature, resting systolic and diastolic blood pressure and pulse including respiratory rates at a supine position after at least 5 min of resting

^4^*Performance status* will be measured using Karnofsky or Lansky scales

^5^Part of clinical routine, see Kontny et al. 2016, Colevas et al. 2018

^6^Serum or urine within 24 h prior to start of chemotherapy

^7^CBC w/differential, (albumin if clinically indicated), LFTs (ALT, AST, total bilirubin, alkaline phosphatase), BUN or serum urea level, creatinine, Ca, Mg, Na, K, LDH, phosphate, glucose, amylase, lipase, cholesterol, triglycerides, uric acid, TSH, Free T4, Free T3. Cholesterol, triglycerides uric acid, TSH, Free T4, Free T3 are not required at visits 2–4 if within normal limits at baseline

^8^Hepatitis B surface antigen (HBV sAg), and hepatitis C antibody (HCV Ab) or hepatitis C RNA (HCV RNA)

^9^Centrally done at Institute of Human Genetics via Laboratory of Pediatric Hematology/Oncology, Uniklinik RWTH Aachen, only for patients receiving 5‑fluorouracil

^10^Centrally done at Institute of Virology, MH Hannover

^11^Initial specimen for EBV-PCR and biobanking can also be obtained before day +1, if informed consent has been obtained. Specimen should be sent that they do not arrive on weekends or holidays

^12^Centrally stored at Uniklinik RWTH Aachen via Laboratory of Pediatric Hematology/Oncology

^13^*MRI* of the head and neck without AND with gadolinium contrast, up to 21 days before start of chemotherapy

^14^(^14^F)-FDG-PET, including diagnostic CT-thorax for detection of pulmonary metastases as part of routine staging

^15^*MRI abdomen* for detection of liver metastases as part or routine staging; in adults, CT-abdomen can also be used, either as separate exam or as diagnostic CT as part of a PET-CT

^16^*AEs* may be volunteered spontaneously by the patient, or discovered as a result of general non-directed questioning by the study personnel or by physical examination. All AEs will be followed until the event resolves or stabilizes at a level acceptable to the investigator

^17^Only at 2 years after enrolment

^18^Audiometry at visits 3 and 4 can be done within 7 days prior to chemo cycles 2 and 3

^19^Day 1 of a chemotherapy cycle is the day when the chemotherapy is started, day 0 is the day preceding, which is up to the local center often used to start hydration

### Sample size {14}

Based on extrapolation of data from the *Landeskrebsregister Nordrhein-Westfalen*, accounting for the incidence of NPC in different age groups and for inclusion of only lymphoepithelial, EBV-positive subtype in adults, as well as considering rates of non-eligibility for the trial in different age groups, the following age distribution of patients eligible for the trial is expected: age 3–17 years: 24% of patients (median age 13 years); age 18–29 years: 9%; age 30–39 years: 10%; age 40–49 years: 22%; age 50–59 years: 19%; age 50–69 years: 16% of patients.

The study is planned according to a two-stage Simon optimum design with alpha = 0.1 and beta = 0.2. With a response rate p0 of 12% for the historical control and an expected better response rate p1 of 25%, 18 patients have to be treated in the first phase (upper limit for rejection: less than three responders). Overall, 48 patients have to be treated in phase I and II when the second stage will be reached (upper limit for rejection: less than nine responders). A dropout rate of five patients who will enter the trial but will not be available for response evaluation due to, i.e., violation of the treatment protocol or insufficient imaging studies is estimated based on the previous *NPC-2003* trial of the GPOH.

Accounting for these statistical considerations, 57 patients in total are planned for enrolment, and screening is expected to be necessary in 65 patients.

### Recruitment {15}

All participating centers are instructed in detail through initiation video conferences. Potential study sites at pediatric and adult oncology centers, radiation therapy departments as well as ear, nose and throat (ENT) specialists, are addressed and regularly reminded of the trial by presentations on conferences and e‑mail newsletters. An information flyer has also been created to address oncologists, radiation oncologists and ENT physicians in Germany to support accrual of patients of all age groups. The study is also advertised through the website of the GPOH, the German Clinical Trials Register (DRKS), and ClinicalTrials.gov. Detailed information and an updated list of open centers are officially available at npc@ukaachen.de.

## Assignment of interventions: allocation

### Sequence generation {16a}, concealment mechanism {16b}, and implementation {16c}

Enrolled patients are allocated to one of five treatment strata based on age, presence or absence of initial distant metastases, and response to induction therapy (see above). Treatment allocation has to be undertaken at two time points, at baseline (visit 2) and after response evaluation (visit 5), and is documented on the respective CRFs.

## Assignment of interventions: blinding

### Who will be blinded {17a}

n/a: As this trial contains no blinded intervention, this section does not apply.

### Procedure for unblinding if needed {17b}

n/a: As this trial contains no blinded intervention, this section does not apply.

## Data collection and management

### Plans for assessment and collection of outcomes {18a}

There will be a screening visit (visit 1) before study enrolment in which patients and/or legal/parent guardians will be informed about the trial, and patients are selected after assessment of inclusion and exclusion criteria. After inclusion in the trial, a detailed clinical and laboratory baseline assessment will be performed as described in the study protocol (visit 2).

Initial imaging studies for staging include MRI of the head/neck, PET-CT/-MRI with diagnostic CT, and MRI of the abdomen. Response assessment after induction therapy will be done by MRI of the head/neck and FDG PET on day 17–22 after the start of the third chemotherapy cycle (visit 5); in patients with distant metastases, imaging studies of metastases using the same methodology as at initial diagnosis will be done as well. In patients with non-metastatic disease and SD or PD after induction therapy as well as in patients with initial distant metastases, treatment with nivolumab is continued during the period of CCRT for a total of six (or seven) doses. These patients will have an additional visit after the end of CCRT (visit 5add), that will take place 12 weeks after the last radiotherapy session and will contain the same measurements and investigations as outlined for visit 5. Radiologic evaluation of tumor response is done by independent central review at the study’s reference radiology and reference nuclear medicine center using the RECIST 1.1 criteria. Image acquisition guidelines and submission process are outlined in the appendices of the study protocol.

After visit 6, patients are further followed up every 3 months until 2 years from study inclusion. The visit schedule with assessments at each visit is depicted in Table 2. Guidelines for measurement of vital signs, collection of blood and saliva, imaging studies, and cardiologic and audiometric evaluation are described in detail in the full version of the study protocol. Furthermore, patient-reported outcomes (PROs) will be assessed using several standardized questionnaires at specified time points (see Table 2). Details on age-specific questionnaires used in the PROs assessment schedule can be found in the appendix of the study protocol.

Safety assessment will consist of monitoring and recording all AEs and SAEs, as defined in the study protocol. All AEs reported by the patient or detected by the investigator will be collected during the trial and must be documented on the appropriate pages of the CRF. All AEs must also be documented in the patient’s medical records. If an AE is assessed as serious, it must be documented in the SAE reporting form additionally, and then submitted per fax to the GPOH center in Essen within 24 h of first becoming aware of the event. This applies regardless of whether, in the opinion of the examiner, there is a causal relationship.

### Plans to promote participant retention and complete follow-up {18b}

n/a: No specific measures have been defined for this purpose. In the case of premature termination of study treatment or active withdrawal of consent, the patient’s study participation is terminated. Return to standard medical care should be ensured. In addition, patients should be encouraged to be registered in the NPC-2016 registry after termination of the study, to obtain further follow-up information.

### Data management {19}

All data to be collected as per the protocol will be entered by the responsible investigator on paper-based CRFs. Transfer of paper-based CRFs into an electronic data collection system will be done by the study data manager. Plausibility checks will be performed to ensure correctness and completeness of the data. After all the data have been entered and all queries have been solved, the database will be closed.

### Confidentiality {27}

All relevant trial data will be stored electronically and handled confidentially. For the statistical analysis and documentation, patients will be identified only by the pseudonymized ID. The investigators and all members of a trial center or other persons involved in the trial are obliged to keep trial data and information confidential and to grant access only to individuals who are involved in the trial. An exception to this rule applies only to representatives of the sponsor or regulatory authorities. All legal requirements concerning the safety and confidentiality of data and prevention of data loss will be respected. All involved individuals are sworn to secrecy. Access to data is strictly limited to authorized persons. Pseudonymization of data in the scope of biometrical analyses is guaranteed.

### Plans for collection, laboratory evaluation, and storage of biological specimens for genetic or molecular analysis in this trial/future use {33}

Laboratory studies will focus on the analysis of biomarkers that could potentially predict clinical response to nivolumab in combination with chemotherapy. Data from these investigations will be evaluated for associations with response, survival (OS, EFS), and/or safety (AE) data. All samples collected may also be used for future exploratory analyses (unless restricted by local requirements) to assess biomarkers associated with NPC or immunotherapy treatment.

At the initial diagnosis (visit 1), tumor tissue is sent for central review to reference pathology (CV), either as one or two blocks (preferred) or a minimum of 25 slides. Additionally, at visit 1, blood is sent for genetic dihydropyrimidine dehydrogenase (DPD) deficiency testing prior to the first 5‑FU exposure to evaluate the risk for excessive drug toxicity and necessity for dose adaptation. Blood will be sent to a reference virology center for EBV polymerase chain reaction (PCR) at different time points to evaluate response of EBV viremia to treatment. Tumor tissue, blood/serum, and saliva will also be sent to the pediatric research laboratory and stored in the biomaterial bank in Aachen, Germany, to enable further molecular analyses on biomaterial in the future.

At each trial visit, a detailed laboratory assessment of the participant is made. For details regarding laboratory studies at each visit, please refer to the visit schedule as depicted in Table 2. Instructions on the collection, handling, and shipment of all samples described herein are provided on the respective shipment forms for reference pathology, reference virology, and biomaterial.

## Statistical methods

### Statistical methods for primary and secondary outcomes {20a}

The safety and efficacy of nivolumab in combination with cisplatin and 5‑FU will be assessed in relation to prior published historical control group data, as a fully powered randomized comparison was not deemed feasible due to the rarity of the patient population. The primary efficacy endpoint is tumor response on MRI and PET-CT/-MRI according to RECIST 1.1 criteria after three cycles of induction therapy. The response rate has been shown to improve in a comparable setting in patients with NSCLC from 29% to 55% [13], and thus an increase from 12% to 25%, which is considered relevant for the patients, seems to be possible. With the assumption of a response rate p0 of 12% for the historical control and an expected better response rate p1 of 25%, 18 patients have to be treated in the first phase of this study with an optimum Simon’s two-stage design with alpha = 0.1 and beta = 0.2 (upper limit for rejection: less than three responders). Overall, 48 patients have to be treated in phase I and II when the second stage will be reached (upper limit for rejection: less than nine responders). As stated in the section “Sample size,” a dropout rate of five patients is estimated. If the study must be stopped after the first phase or the response rate fails to exceed the level of historical control (less than nine responders at the second stage), nivolumab will not be considered an active agent. All patients with evaluable response who received at least one dose of nivolumab will be analyzed for the primary outcome.

As secondary endpoints, EFS and OS will be analyzed with suitable descriptive methods (Kaplan–Meyer estimates with confidence intervals) and compared with historical data using descriptive log-rank tests. As almost all events occur within 2 years of the diagnosis, a follow-up period of 2 years is considered sufficient [8]. All patients who received at least one dose of nivolumab will be analyzed for safety with descriptive methods (frequency tables, rates of AEs and severe adverse events [SAEs] with 95% confidence intervals).

### Interim analyses {21b}

An interim analysis will be conducted after response data for 18 patients are available. If there are less than three responders, the study hypothesis, an expected better response rate p1 of 25% versus a response rate p0 of 12%, is rejected and the study will be prematurely closed. If there are three or more responders, the study enters the second phase, enrolling a total of 48 patients for response evaluation.

#### Methods for additional analyses (e.g., subgroup analyses) {20b}

If indicated, additional data will be analyzed with appropriate descriptive methods.

#### Methods in analysis to handle protocol non-adherence and any statistical methods to handle missing data {20c}

Non-adherence to the study protocol will be described in the final analysis, if indicated. Missing data will not be considered in the final analysis.

#### Plans to give access to the full protocol, participant level-data, and statistical code {31c}

The trial will remain open until 2 years after the date of the last patient’s first visit. The sponsor will notify the relevant Competent Authority and Ethics Committee that the trial has ended at the appropriate time and will provide them with a summary of the clinical trial report within 12 months of the end of trial. Study results will be published in appropriate international scientific journals, and publishing details will be given in the clinical study agreement. The study will be registered and study results, together with the study protocol and the statistical analysis plan, will be disclosed by the principal investigator in one or more public clinical study registry(ies), according to national/international use.

## Oversight and monitoring

### Composition of the coordinating center and trial steering committee {5d}

Study coordination, data management, monitoring, and safety management will be done by the Pediatric Research Network gGmbH, University of Duisburg-Essen, which has comprehensive experience in management and monitoring of pediatric drug trials (leading project management by HK). The expert steering committee (HC, THB, MJE, TF, J‑PK, FMM, BT, MZ, UK) will oversee the trial and regularly discuss protocol recommendations by considering the most recent scientific evidence.

### Composition of the data monitoring committee, its role, and reporting structure {21a}

To provide independent trial monitoring, a qualified data monitoring committee from the GPOH is established, which will regularly monitor the study according to good clinical practice (GCP) guidelines and the respective standard operating procedures (SOPs). The monitors are trained during a monitoring kick-off meeting. Monitoring procedures include one or more visits designed to clarify all prerequisites before the study commences. Interim monitoring visits will take place on a regular basis according to a mutually agreed schedule. During these visits, the monitor will check for completion of the entries on the CRF; for compliance with the clinical study protocol, ICH-GCP principles, the Declaration of Helsinki, and regulatory authority requirements; for the integrity of the source data with the CRF entries; and for participant eligibility. Monitoring also will be aimed at detecting any misconduct or fraud. In addition, the monitor will check whether all AEs and SAEs have been reported appropriately within the time periods required. The investigators and all staff are expected to cooperate with the data monitoring committee by providing any missing information whenever possible and answering questions arising during regular monitoring visits. All participants who give their informed consent, including those screened but not entered into the study, will be listed on the participant screening/enrolment log. Further details of monitoring activities are set forth in a monitoring manual.

### Adverse event reporting and harms {22}

Safety assessment will consist of monitoring and recording all AEs and SAEs, the regular monitoring of hematology, blood chemistry and urine values, audiometry, regular measurement of vital signs, and the performance of physical examinations.

An AE is defined according to the ICH Guideline for GCP (ICH E6:1.2). The grading of AEs will be carried out based on the 5‑grade scale defined in the CTCAE v5.0. Grading of AEs not listed in the CTCAE v5.0 will be done by the responsible investigator and classified as mild, moderate, or severe as per protocol definitions. Medical judgment should be used to determine causal relationship of AEs, considering all relevant factors, including pattern of reaction, temporal relationship, de-challenge or re-challenge, confounding factors such as concomitant medication, concomitant diseases, and relevant history. Assessment of causal relationship must be recorded for each AE. The SAEs are defined according to the “Detailed guidance on the collection, verification and presentation of adverse event/reaction reports arising from clinical trials on medicinal products for human use—2011/C 172/01 CT-3” and Article 2 (33) of EU Regulation 536/2014. Please refer to the full protocol for study-specific considerations regarding SAEs. All AEs reported by the patient or detected by the investigator will be collected during the trial and must be documented on the appropriate pages of the CRF. If an AE is assessed as serious, it must be documented in the SAE Report Form additionally. This applies regardless of whether, in the opinion of the examiner, there is a causal relationship. The investigator is responsible for ensuring that all SAEs observed by the investigator or reported by the participant that occur after the beginning of the study treatment through 100 days after the last dose of the study treatment are recorded in the participant’s medical record and are submitted within 24 h of first becoming aware of the event per fax to the sponsor.

### Frequency and plans for auditing trial conduct {23}

Audits will be performed according to the corresponding audit program, including the possibility that a member of the sponsor’s quality assurance function may arrange to visit the investigator to audit the performance of the study at the study site, as well as all study documents originating there. Auditors conduct their work independently of the clinical study and its performance. In the case of audits at the investigational site, the monitor will usually accompany the auditor(s). For further data monitoring aspects, please see section {21a}.

### Plans for communicating important protocol amendments to relevant parties (e.g., trial participants, ethical committees) {25}

Any protocol modifications are communicated to relevant parties including investigators and regulators. Substantial amendments that require approval according to the national GCP regulation will be submitted to the responsible authorities for evaluation, and are only implemented after their approval.

## Dissemination plans {31a}

The trial is registered with full description at the DRKS, and at ClinicalTrials.gov by the United States National Library of Medicine. Study results will be published in appropriate national and/or international scientific journals. Beyond regular journal publication, results will be presented and discussed at national and/or international congresses addressing scientific authorities involved in the treatment of pediatric and adult patients with NPC. Furthermore, results will be published in the European Union Drug Regulation Authorities Clinical Trials (EudraCT) database. Raw data will be made available in a data repository. To enhance patient interest and support, information in lay language is provided on our homepage https://www.ukaachen.de/kliniken-institute/klinik-fuer-kinder-und-jugendmedizin/klinik/sektionen/paediatrische-haematologie-onkologie-und-stammzelltransplantation/nasopharynxkarzinom-studiengruppe/npc-nivo-studie/informationen-fuer-patienten/. Moreover, we will be in direct contact and interaction with ENT, radiotherapy, and oncology centers in Germany to support decision-making concerning optimal treatment for pediatric and adult patients with NPC, regardless of study participation.

## Discussion

This prospective phase 2 trial aims to assess the benefit of adding the anti-PD‑1 antibody nivolumab to standard induction chemotherapy in patients with EBV-positive NPC, with complete response rate after cisplatin/5-FU plus nivolumab induction as the primary endpoint.

Response-adapted dosing of definitive radiotherapy will possibly reduce the high burden of late toxicities, which is a matter of great importance in a tumor with excellent primary cure rates today. By combining immune checkpoint-inhibition with conventional induction chemotherapy, we hope to increase antitumor efficacy without aggravating acute toxicity.

Regarding the combination of an anti-PD‑1 antibody with conventional chemotherapy in adults, the *Keynote-048* trial showed a comparable safety profile between the pembrolizumab + cisplatin/5-FU and the cetuximab + cisplatin/5-FU arm [17]. Furthermore, a 5-year safety and efficacy analysis from a phase 1b trial with 24 patients with NSCLC who received nivolumab together with four different chemotherapy regimens (cisplatin/gemcitabine, cisplatin/pemetrexed, carboplatin/paclitaxel, docetaxel) showed no unexpected SAEs or treatment-related deaths [22]. Data on the safety and efficacy of nivolumab in children are available from a large multicenter phase 1/2 trial, the *ADVL1412* trial. In this trial, nivolumab was given to 85 children and young adults with relapsed or refractory solid tumors. In the dose-confirmation phase of this trial, a dose of 3 mg/kg administered intravenously over 60 min every 2 weeks could be established for children, with no dose-limiting toxicities observed. In the phase 2-part, dose-limiting toxicities were seen in five patients (7%), with cytopenias and fatigue as the most common grade 3 or 4 AEs. Immune-related AEs were hepatic toxicity with transaminitis (about 30%), and pleural or pericardial effusions in 11 patients (15%), of which only four were attributed to study treatment. Overall, AEs led to discontinuation of nivolumab in only 10% of patients, and no death was attributed to study treatment [19]. This trial served as the basis for dose calculation of nivolumab in our trial, as it showed a favorable safety profile and high tolerability for the regimen of 3 mg/kg every 2 weeks. In order to improve adherence to the study protocol by synchronizing the dosing intervals of nivolumab and chemotherapy, we chose a dose regimen of 4.5 mg/kg every 3 weeks, with a capping dose of 360 mg that had been approved in adults for gastric and esophageal carcinoma in combination with fluoropyrimidine- and platinum-based chemotherapy [23]. Furthermore, a phase 3 trial in adults with esophageal squamous cell carcinoma has investigated the combination of nivolumab 240 mg every 2 weeks plus cisplatin/5-FU every 4 weeks, also showing good tolerability [24]. As the regimens of 3 mg/kg every 2 weeks and the 6 mg/kg every 4 weeks in children and adolescents have shown a similar exposure profile as the tolerated regimen of 10 mg/kg every 2 weeks in adults, exposures with a regimen of 4.5 mg/kg every 3 weeks are predicted to lie within the tolerated range. Although a dose simulation for this regimen has not formally been made, exposure differences between pediatric and adult patients are expected to fall between those at 3 mg/kg every 2 weeks and 6 mg/kg every 4 weeks. Therefore, we expect this dosing regimen to be safe and feasible in children as in adults, with the potential to increase adherence to the study protocol by reduction of treatment sessions. It, nevertheless, must be kept in mind that the aforementioned considerations are made in the context of monotherapy with nivolumab. By combining nivolumab with cisplatin/5-FU, any pharmacokinetic or pharmacodynamic interactions, especially overlapping side effects, must be considered. However, based on data from adults, we do not expect a clinically meaningful interaction between the medical components or any new safety aspects. Published evidence for the combination of nivolumab with CCRT in children is lacking to date, but as for the combination with chemotherapy and based on data from adults, we expect it also to be safe and feasible [25–27]. Anyway, safety analysis of the combination of nivolumab with induction chemotherapy and CCRT will be an important outcome parameter of our trial. To account for this safety aspect, we applied stringent criteria for dose modifications or delays of nivolumab and chemotherapy separately in our study protocol and developed detailed CRFs for toxicity reporting.

By dichotomizing patients in age groups of ≤ 25 and > 25 years, we aim not only to investigate the effect of additional nivolumab in young patients, but also to assess the beneficial effect on EFS and OS in older patients with locoregionally advanced or metastatic NPC. For children, adolescents, and young adults with locally advanced NPC, the benefit of induction chemotherapy with cisplatin/5-FU has convincingly been demonstrated by the GPOH and other study groups [5–8]. By contrast, the role of induction chemotherapy in adults has long been controversial. However, due to recent randomized trials, induction chemotherapy has proved advantageous also in adults with locally advanced NPC in terms of disease-free survival (DFS) and OS [28–30]. Two well-designed meta-analyses suggest improvement of local tumor control by induction chemotherapy, underpinning its value in adults [31, 32]. The most used induction regimens in adults are docetaxel/cisplatin with or without 5‑FU and gemcitabine/cisplatin. We are aware of the concern for a delay of definitive CCRT due to excessive toxicity after induction chemotherapy in older patients who might have additional morbidities, but data from the above cited studies do not point toward an unacceptable toxicity profile, and our stringent exclusion criteria will prevent inclusion of patients with an unacceptable risk of toxicity. We therefore expect that induction chemotherapy will not hinder timely delivery of subsequent full-dose CCRT. Furthermore, all cited studies investigating induction chemotherapy in adults included only patients with stage III or IV disease, and thus we also restricted inclusion of patients older than 25 years to stage III or IV disease, assuming that these patients will have the most favorable risk–benefit assessment with induction chemotherapy. While patients older than 25 years with non-metastatic disease are scheduled to receive cisplatin/5-FU as induction, patients with metastatic disease will receive gemcitabine/cisplatin, as this regimen has proved superior in this scenario and age group in a randomized phase 3 trial [33]. As adjuvant IFN‑β treatment has been studied mainly in the GPOH studies and experiences in adults are limited, we restrict IFN‑β treatment after CCRT to patients ≤ 25 years of age.

In summary, our trial aims to investigate the beneficial effect and safety of addition of the immune checkpoint-inhibitor nivolumab in the first-line treatment of locoregionally advanced or metastatic EBV-positive NPC both in pediatric and in adult patients. Combination of immunotherapy with conventional chemotherapy or radiotherapy is a promising strategy that is being explored in many ongoing trials in different cancer types. We hope to better define the role of immunotherapy in NPC by exploring its effect on tumor response and survival in this age-spanning trial setting.

## Trial status

- Protocol version: v1.3, March 20, 2023
- First patient enrolled: January 10, 2023
- Estimated recruitment completion: Q1 2025
- Current status: First five patients enrolled

## Data Availability

The investigators and all members of a trial center or other persons involved in the trial are obliged to keep trial data and information confidential and to grant access only to individuals who are involved in the trial. An exception to this rule applies only to representatives of the sponsor or regulatory authorities, who will have full access to all trial data at any time. Relevant trial data will be shared in a pseudonymized form with other physicians and scientists (national and international) in the context of publications to promote and accelerate research in the field of NPC treatment.

## Abbreviations

[^18^F]FDG: 18-fluorodeoxyglucose
AE: adverse event
AJCC: American Joint Committee on Cancer
ALT: alanine aminotransferase
AST: aspartate aminotransferase
CCRT: concomitant chemoradiotherapy
Ch A: chemotherapy cycle A
Ch B: chemotherapy cycle B
Ch C: chemotherapy cycle C
CPS: Combined Positive Score
CR: complete response
CRF: case report form
CT: computerized tomography
CTCAE: common terminology criteria for adverse events
CTIS: Clinical Trials Information System
CTL: cytotoxic T‑lymphocyte
CTLA‑4: cytotoxic T‑lymphocyte-associated antigen‑4
DFS: disease-free survival
DPD: dihydropyrimidine dehydrogenase
ENT: ear, nose and throat
EudraCT: European Union Drug Regulation Authorities Clinical Trials
EBV: Epstein–Barr virus
EFS: event-free survival
GCP: good clinical practice
GPOH: German Society of Pediatric Oncology and Hematology
HNSCC: head and neck squamous cell carcinoma
ICH: International Council for Harmonisation of Technical Requirements for Pharmaceuticals for Human Use
IFN‑β: Interferon beta
IMP: investigational medicinal product
IMRT: intensity-modulated radiotherapy
IU: international units
MRI: magnetic resonance imaging
NCCN: National Comprehensive Cancer Network
NPC: nasopharyngeal carcinoma
NSCLC: non-small-cell lung cancer
OS: overall survival
PCR: polymerase chain reaction
PD: progressive disease
PD‑1: programmed cell death 1
PD-L1: programmed cell death ligand 1
PET: positron emission tomography
PFS: progression-free survival
PR: partial response
PROs: patient-reported outcomes
PTV: primary target volume
RECIST: Response Evaluation Criteria in Solid Tumors
SAE: serious adverse event
SD: stable disease
SOP: standard operating procedure
SPIRIT: Standard Protocol Items: Recommendations for Interventional Trials
ULN: upper limit of normal
WBC: white blood cells
WHO: World Health Organization

## Declarations

This study was first approved on August 8, 2022, by the German national competent authority Paul Ehrlich Institute (PEI) and the Ethics Committee under the EU Clinical Trials Regulation 536/2014 via the Clinical Trials Information System (CTIS). Conduct of the trial including patient information and consent forms were authorized by the European Medicines Agency (No. 2022-500676-59-00). All studies mentioned were in accordance with the ethical standards indicated in each case. Consent for publication of data is integrated in the general consent form, which is handled out initially to the patients and/or parent/legal guardians.
